# Recycling Pricing and Coordination of WEEE Dual-Channel Closed-Loop Supply Chain Considering Consumers’ Bargaining

**DOI:** 10.3390/ijerph14121578

**Published:** 2017-12-15

**Authors:** Xiaodong Zhu, Jing Wang, Juan Tang

**Affiliations:** 1 School of Economics and Management, Nanjing University of Aeronautics and Astronautics, Nanjing 210016, China; zxd@nuist.edu.cn; 2 School of Management Science and Engineering, Nanjing University of Information Science and Technology, Nanjing 210044, China; 3 School of Management, Huazhong University of Science and Technology, Wuhan 430070, China; j_w@hust.edu.cn; 4 College of Management Engineering, Anhui Polytechnic University, Wuhu 241000, China

**Keywords:** closed-loop supply chain, dual-channel recycling, bargaining behavior, pricing mechanism, revenue and expense sharing contract

## Abstract

Environmentally friendly handling and efficient recycling of waste electrical on Waste Electrical and Electronic Equipment (WEEE) have grown to be a global social problem. As holders of WEEE, consumers have a significant effect on the recycling process. A consideration of and attention to the influence of consumer behavior in the recycling process can help achieve more effective recycling of WEEE. In this paper, we built a dual-channel closed-loop supply chain model composed of manufacturers, retailers, and network recycling platforms. Based on the influence of customer bargaining behavior, we studied several different scenarios of centralized decision-making, decentralized decision-making, and contract coordination, using the Stackelberg game theory. The results show that retailers and network recycling platforms will reduce the direct recovery prices to maintain their own profit when considering the impact of consumer bargaining behavior, while remanufacturers will improve the transfer payment price for surrendering part of the profit under revenue and the expense sharing contract. Using this contract, we can achieve supply chain coordination and eliminate the effect of consumer bargaining behavior on supply chain performance. It can be viewed from the parameter sensitivity analysis that when we select the appropriate sharing coefficient, the closed-loop supply chain can achieve the same system performance under a centralized decision.

## 1. Introduction

As the electrical and electronic products of the last generation become obsolete, and upgrades of electronic products accelerate, the total number of China’s waste electrical and electronic equipment (WEEE) continues to increase. In 2016, there were approximately 75 million units of WEEE accounted for by 109 subsidized enterprises that processed WEEE. By 2020, based on a 20% growth rate, there will be 137 million units of electronic waste products [1]. As a recyclable resource, electronic waste products and related parts have a high recycling value. However, there are many challenges involved, and the handling costs are high. In addition, waste products and parts contain high levels of toxic materials, and are the major source of urban heavy metal pollution, which, if not properly treated, will lead to a huge waste of resources, as well as further damage the global ecosystem. As the recycling and reuse of waste products becomes popular, a closed-loop supply chain (CLSC) is created. Structurally, CLSC is a closed system created by combining the traditional forward and reverse logistics that has emerged in recent years. In a 1992 report submitted to the Council of Logistics Management (CLM) in the United States, the concept of reverse logistics was first put forward by Stock, who argued that all related logistics activities involved in raw material savings, recycling and reuse, substitution, material recycling, and disposal are organically combined, and impact one another. CLSC management is a comprehensive approach that deals with the recycling, handling, remanufacturing, and resale of waste and used products to appropriately and effectively allocate existing resources, protect the existing ecological environment, and provide a realistic approach to achieve sustainable development [2]. Implementing CLSC lowers the production costs of enterprises, reduces loss in social resources, creates social benefits, and enhances the rapid development of economic benefits from electronic waste products. However, effective CLSC management will only be achieved if manufacturers, retailers, consumers, the government, and other major constituents work together. In the Internet age that we are in, many enterprises have taken advantage of new development opportunities, and have built their own online recycling platforms using internet-based information technology to expand electronic waste recycling channels and increase recycling rates. “Jindong Recycling Network” is a typical example of offline recycling using an internet platform. The actual recycling process starts with an enterprise that conducts a simple survey on the quality of products available online that the enterprise is interested in recycling, and price estimates will then be provided based on the survey results. When consumers participate in the offline transaction, they negotiate on the price offered by the enterprise. In other words, the prices set by the enterprise are not necessarily the final transaction price. There is room for negotiation because the online price estimated by enterprise may have underestimated the product quality. In addition, there are no industry standards or national systems for recycled product pricing. The bargaining power of consumers on the pricing of waste and used electronic products affects the decision-making behavior of the related decision-making system. Thus, it is of practical and theoretical importance to incorporate the impact of consumer bargaining power on multi-system behavior when studying supply chain pricing and coordination.

Many scholars have conducted comprehensive studies on various fundamental areas of CLSC such as its recycling system [3,4], laws and regulations [5,6], and online recycling planning [7,8]. Other related studies have examined CLSC recycling channel selection, recycling pricing and supply chain coordination, and consumer behavior, etc. In terms of recycling channel selection, Feng [9] adopted Stackelberg game theory to study the issues of a dual-hierarchical reverse supply chain in a dual recycling channel, and found that it is better than the single-channel, at least from the distributor’s and systemic perspective. Feng [9] also suggested using customs contracts and profit-sharing contracts in reverse supply chain system coordination. Using the single-channel and dual-channel supply chain scenarios, George [10] studied the impact of channel structure and coordination on suppliers, retailers, and the overall supply chain. By comparing the supply chain performance of single-channel recycling and dual-channel recycling, Huang [11] pointed out that dual-channel recycling has a stronger comparative advantage, and put forth the optimal CLSC strategy in dual-channel recycling using game theory. In terms of CLSC pricing and coordination, Amaro et al. [12] designed a comprehensive and complex CLSC network by combining forward and reverse supply chains. Considering market demand and price, as well as uncertainties among other supply chain conditions, a multi-period programming model was constructed using mixed integer linear programming (MILP) to identify the optimal solution. Savaskan et al. [13] examined the reverse channel structure and pricing strategy in three different models with manufacturers, retailers, and a third-party carrying out recycling, respectively. Wu [14] constructed a two-period game model to calculate the relationship between a product design strategy and pricing strategy, which was then extended into a multi-period model. Based on social welfare maximization, Wang [15] constructed a Stackelberg social welfare game model to determine recycling pricing and the maximization of social subsidy in reverse logistics under decentralized CLSC management. Based on a two-stage reverse logistics model consisting of one manufacturer and one retailer, Heydari [16] analyzed the functions of various government incentive measures (tax-free and subsidies) provided to supply chain players to incentivize logistics coordination. In terms of consumer characteristics, Bakal [17] examined the recycling and remanufacturing activities of one single enterprise based on random needs and consumer price sensitivity using a maximization approach to discuss the optimal recycling price and quantity. Debabrata [18] studied the relationship between consumer incentives and the return rate of products faced by retailers, and confirmed that quotation incentives enhance system profits and the replenishment rate. Based on consumer disposal behavior and recycling awareness [19,20] analyzed their positive role in the related country’s strategy formation in electronic waste management. Liu et al. [21] applied a stylized model to characterize the optimal solutions for the manufacturer and analyzed the effects of the fund policy on the manufacturer’s collection and recovery decisions. Li et al. [22] studied optimal operation in the competitive pricing, competitive the low-carbon promotion, the carbon emission reduction, the used-products collection and the profits. Chen et al. [23] explored the dual PV supply chain competition under the Bertrand competition assumption by three game-theoretical modeling scenarios considering either the public subsidy or no subsidy from a social welfare maximization perspective. Huang et al. [24] explores the optimal strategies for a retailer-dominated closed-loop supply chain (CLSC) with a triple recycling channel in the construction machinery remanufacturing context. Ma et al. [25] studied the sensitive relationship between consumers’ environmental awareness level and the direct recycling payment price, and found that the higher the environmental awareness and the more sensitive to WEEE recycling price, the more conducive the two conditions are to increase the WEEE recycling quantity that is returned to enterprises. Literature [26,27] around WEEE as an example to carry out related research. Xie et al. [28] studies contract coordination of centralized and decentralized dual-channel closed-loop supply chains.

Electronic waste recycling and handling is a well-studied research topic. We can see from the past literature that the primary focus has been on two perspectives, qualitative and quantitative. Most of the qualitative studies have focused on various areas of WEEE recycling such as laws and regulations, the recycling and handling systems, and funding mechanisms, etc. On the other hand, most of the quantitative studies have focused on model construction related to recycling channel selection, recycling pricing, and supply chain coordination, etc. Little research has focused on consumer bargaining power in the supply chain. This study considers the emergence of the internet as a contextual background, and incorporates consumer bargaining power into the recycling supply chain to study the issues of recycling pricing and supply chain coordination in dual-channel electronic waste recycling. A dual-channel recycling mechanism is a new concept introduced in the current internet era that is based on one single major recycling system combined with an additional recycling channel (i.e., recycling takes place via a third-party recycling platform). This study starts with the dual-channel electronic waste recycling model, incorporates consumer bargaining power to a certain extent during recycling to determine the optimal recycling pricing, and analyzes the coordination mechanism so that profit maximization is achieved in each enterprise node of the supply chain and systemic efficiency is promoted under a decentralized condition.

Subsequent sections of the paper are organized as follows. In the second section, the adopted model is described, and the assumptions used are explained. The third section focuses on constructing a CLSC model under both centralized and decentralized decision-making scenarios, and analyzes it, identifying its solutions. The fourth section discusses profit coordination among three main players, i.e., manufacturers, retailers, and recycling platforms, in the supply chain by a contract mechanism design. In the fifth section, a numerical case study is presented based on the models and resolutions are identified to verify related findings of the study. Finally, conclusions are presented in the sixth section.

## 2. Description of the Problem and Related Assumptions

### 2.1. Description of the Problem

**Figure 1:**
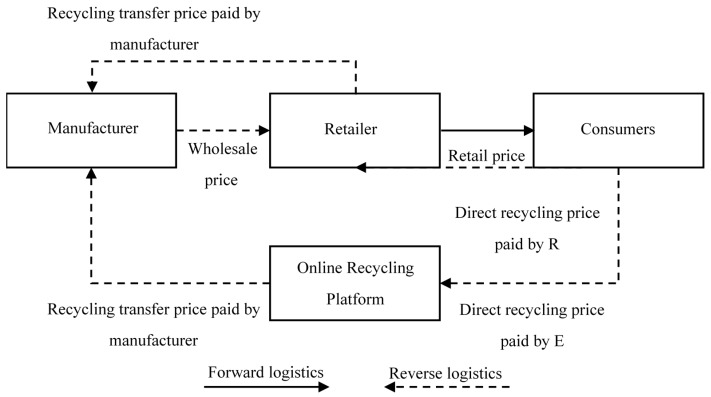
The CLSC dual-channel recycling model.

Traditional recycling channels include retailers carrying out recycling and manufacturers carrying out direct recycling, etc. In this study, an online recycling channel is introduced to construct a dual-channel recycling model between retailers and a third-party online recycling platform. Within this context, manufacturer M is the primary leader in the supply chain who manufactures new products and sells wholesale to retailers at the unit wholesale price. The manufacturer also determines the unit recycling transfer price paid to retailer R, and to third-party online recycling platform E for subsequent electronic waste recycling and remanufacturing. Retailer R sells the new products and also competes with the third-party online recycling platform E in the electronic waste market. Based on the unit recycling transfer pricing set by the manufacturer M under the principle of profit maximization, direct recycling pricing is determined by retailer R and the third-party online recycling platform E, individually. The model is illustrated in Figure 1 below.

### 2.2. Related Assumptions

**Assumption** **1.** *Assume that the outlook, quality, and functions are the same between remanufactured and new products (i.e., product quality is homogeneous) [7]. Parts disassembled from electronic waste products are repaired by technicians that satisfy normal usage standards such that product quality and functions of the remanufactured product made from the components of electronic waste are the same as in new products made from brand new materials. Therefore, both types of products enter the market alike, and are for sale at the same retail price*.

**Assumption** **2.** Assume that the impact of consumer bargaining power ∅ on the recycling price of electronic waste is linear, and the higher the consumer bargaining power is, the higher the recycling price is. The recycling price is represented as a direct recycling payment, A*(1+∅) , where ∅∈[0,1]. Considering channel competition exists in the dual-channel, the relationship between the recycling price and quantity is illustrated as follows, where $m_{0}$ represents the voluntary market recycling quantity when recycling price = 0, a consumer’s voluntary waste recycling quantity is $m_{1}$ and $m_{2}$ represents the consumer’s sensitivity coefficient to the recycling channel price.

$$q_{R}=m_{0}+m_{1}A_{R}\left(1+\emptyset \right)+m_{2}\left(1+\emptyset \right)\left(A_{R}-A_{E}\right),$$

$$q_{E}=m_{0}+m_{1}A_{E}\left(1+\emptyset \right)+m_{1}\left(1+\emptyset \right)\left(A_{E}-A_{R}\right)$$

**Assumption** **3.** *Assume that the coefficient of market demand for new and remanufactured products is expressed in a linear equation, D(p)=α−βp, where β is the coefficient of the consumer price sensitivity and α is the potential market size*.

**Assumption** **4.** *Assume that manufacturer M’s unit cost of producing a new electronic product is $c_{1}$, which is greater than the unit cost of remanufacturing $c_{2}$ from recycled electronic parts, that is, M’s unit cost savings during electronic waste recycling are $\mu =c_{1}-c_{2}\geq 0$. Assume that the retailer’s demand for new products at the price set by M based on production costs is greater than zero, that is, $\alpha -c_{1}\beta >0$. Assume that all parties of the recycling process participate in recycling based on willingness, that is, the channel recycling transfer price paid by M is greater than the cost of direct recycling paid to consumers, and the recycling transfer price paid by M is smaller than the cost savings from remanufacturing. Hence, all parties involved willingly participate in recycling, that is, $A\leq {Β}_{R}\leq \mu A\leq {Β}_{E}\leq \mu$*.

**Assumption** **5.** *Assume that the market structure examined in this study is led by manufacturer M, that is, manufacturer M has full channel rights, and each enterprise node makes decisions based on their own profit maximization. Assume also that all parties are under complete information symmetry*.

The symbols used in this paper are summarized in Table 1.

## 3. Model Construction and Analysis

### 3.1. Decentralized Dual-Channel Recycling Model Analysis

Dual-channel recycling decision-making model:

(1) $$\{\begin{array}{c} \underset{w,B_{R},B_{E}}{\max}\Pi_{M}^{D}=wD\left(p\right)+\mu \left(q_{R}+q_{E}\right)-B_{R}q_{R}-B_{E}q_{E}\\ s.t.\{\begin{array}{c} \left(p,A_{R}\right)\in \arg max\Pi_{R}^{D}=\left(p-w\right)D\left(p\right)+B_{R}q_{R}-q_{R}A_{R}\left(1+\emptyset \right)\\ A_{E}\in \arg max\Pi_{E}^{D}=\left(B_{E}-\left(1+\emptyset \right)A_{E}\right)q_{E} \end{array} \end{array}$$

Manufacturer’s profit function is:

$$\Pi_{M}^{D}=(w-c_{1})\left(\alpha -\beta p\right)+\mu \left(q_{R}+q_{E}\right)-B_{R}q_{R}-B_{E}q_{E}$$

Retailer’s profit function is:

$$\Pi_{R}^{D}=\left(p-w\right)\left(\alpha -\beta p\right)+B_{R}q_{R}-q_{R}A_{R}\left(1+\emptyset \right)$$

Online recycling platform’s profit function is:

$$\Pi_{E}^{D}=\left(B_{E}-\left(1+\emptyset \right)A_{E}\right)q_{E}$$

In a decentralized decision-making model, the manufacturer is the game leader, whereas the retailer and third-party online recycling platform are followers and competitors of each other who make decisions simultaneously. We use reverse regression to resolve the optimal decision-making strategy of each player in the game.

By solving the first-order partial derivative of p and $A_{R}$ in $\Pi_{R}^{D}$ and solving the first-order partial derivative of $A_{E}$ in $\Pi_{E}^{D}$, subject to $\frac{\partial \Pi_{R}^{D}}{\partial p}=0,\frac{\partial \Pi_{R}^{D}}{\partial A_{R}}=0,\frac{\partial \Pi_{E}^{D}}{\partial A_{E}}=0$, we obtain the following:

$$p=\frac{\alpha +w\beta }{2\beta }$$

$$A_{R}=\frac{-m_{0}\left(2m_{1}+3m_{2}\right)+\left(m_{1}+m_{2}\right)\left(B_{R}m_{2}+2B_{E}\left(m_{1}+m_{2}\right)\right)}{\left(4m_{1}{}^{2}+8m_{1}m_{2}+3m_{2}{}^{2}\right)\left(1+\emptyset \right)}$$

$$A_{E}=\frac{-m_{0}\left(2m_{1}+3m_{2}\right)+\left(m_{1}+m_{2}\right)\left(B_{R}m_{2}+2B_{E}\left(m_{1}+m_{2}\right)\right)}{\left(4m_{1}{}^{2}+8m_{1}m_{2}+3m_{2}{}^{2}\right)\left(1+\emptyset \right)}$$

We substitute the above equation into $\Pi_{M}^{D}$, solve the first-order partial derivative of $w,\;B_{R}$, and $B_{E}$ in $\Pi_{M}^{D}$, subject to $\frac{\partial \Pi_{M}^{D}}{\partial w}=0,\frac{\partial \Pi_{M}^{D}}{\partial B_{R}}=0,\frac{\partial \Pi_{M}^{D}}{\partial B_{E}}=0$, and we obtain, $w=-\frac{-\alpha -c_{1}\beta }{2\beta },\;B_{E}=-\frac{m_{0}-m_{1}\mu }{2m_{1}},\;B_{R}=-\frac{m_{0}-m_{1}\mu }{2m_{1}}$.

We substitute the above equation back into $p,\;A_{R},\;A_{E},$ and we obtain, $p^{D*}=\frac{3\alpha +2c_{1}\beta }{4\beta },\;A_{E}^{D*}=A_{R}^{D*}=\frac{-m_{0}\left(3m_{1}+m_{2}\right)+m_{1}\left(m_{1}+m_{2}\right)\mu }{2m_{1}\left(2m_{1}+m_{2}\right)\left(1+\emptyset \right)}$.

We substitute each optimal solution into the profit coefficient, and obtain the following profit optimization for the manufacturer, retailer, and online recycling platform, respectively:

$$\Pi_{M}^{D*}=\frac{X}{8m_{1}\left(2m_{1}+m_{2}\right)\beta },\;\Pi_{R}^{D*}=\frac{Y}{16{\left(2m_{1}+m_{2}\right)}^{2}\beta },\;\Pi_{E}^{D*}=\frac{\left(m_{1}+m_{2}\right){\left(m_{0}+m_{1}\mu \right)}^{2}}{4{\left(2m_{1}+m_{2}\right)}^{2}}.$$

The details of simplification see Appendix A.

**Proposition** **1.** *Under a decentralized decision-making scenario, $w=\frac{\alpha +c_{1}\beta }{2\beta },\;{Β}_{E}={Β}_{R}=-\frac{m_{0}-m_{1}\mu }{2m_{1}}$, is the manufacturer’s optimal pricing strategy; $p=\frac{\alpha +\frac{1}{2}\left(\alpha +c1\beta \right)}{2\beta }$, $A_{R}=\frac{-m_{0}\left(3m_{1}+m_{2}\right)+m_{1}\left(m_{1}+m_{2}\right)\mu }{2m_{1}\left(2m_{1}+m_{2}\right)\left(1+\emptyset \right)}$, is the retailer’s optimal pricing strategy; and $A_{E}=\frac{-m_{0}\left(3m_{1}+m_{2}\right)+m_{1}\left(m_{1}+m_{2}\right)\mu }{2m_{1}\left(2m_{1}+m_{2}\right)\left(1+\emptyset \right)}$ is the online recycling platform’s optimal pricing strategy (Please see Appendix B for proof)*.

Proposition 1 shows that consumer bargaining power only affects the direct recycling price of electronic wastes, and does not affect the recycling transfer payment price, product wholesale price, or sale price. Therefore, manufacturers do not need to consider the impact of consumer bargaining power on product recycling quantity when making decisions on pricing. On the other hand, retailers and third-party online recycling platforms, being direct recycling enterprises, need to consider consumer bargaining power in making decisions to set the optimal recycling price, and achieve profit maximization for the enterprises. From a theoretical point of view, the manufacturer plays a dominant role in the supply chain forming an oligopoly, and has great control on the recycling transfer payment price. Therefore, at stage 1, the manufacturer only needs to formulate the optimal recycling transfer payment price to ensure its own profit maximization. In stage 2, consumer bargaining power will impact the direct recycling price. Being direct recycling enterprises, the retailer and the third-party online recycling platform need to take advantage of the volatility in recycling price to establish an optimal recycling pricing mechanism.

### 3.2. Centralized Dual-Channel Recycling Model Analysis

In a centralized decision-making model, to achieve the optimal performance of the overall supply chain, each main player voluntarily eliminates the impact that results from internal competition. Their decision making is no longer solely based on their own profit maximization, but rather on the overall supply chain. Thus, a unified decision-making system is formed between the manufacturer, retailer, and the online recycling platform, whereby the CLSC system profit function is as follows:

(2) $$\Pi_{S}^{C}\left(p,A\right)=\left(p-c_{1}\right)\left(\alpha -\beta p\right)+\left[\mu -A\left(1+\emptyset \right)\right]\left(q_{E}+q_{R}\right)$$

By solving Equation (2) based on profit maximization, we have:

$$\frac{\partial \Pi_{S}^{C}}{\partial p}=\alpha -p\beta -\left(-c_{1}+p\right)\beta$$

$$\frac{\partial \Pi_{S}^{C}}{\partial A}=\left(-1-\emptyset \right)\left(2m_{0}+Am_{1}\left(1+\emptyset \right)\right)+m_{1}\left(1+\emptyset \right)\left(-A\left(1+\emptyset \right)+\mu \right)$$

Subject to $\frac{\partial \Pi_{S}^{C}}{\partial p}=0,\;\frac{\partial \Pi_{S}^{C}}{\partial A}=0$, we have:

$$p^{C*}=\frac{\alpha +c_{1}\beta }{2\beta },A^{C*}=\frac{-2m_{0}+m_{1}\mu }{2m_{1}\left(1+\emptyset \right)}$$

The optimal profit of the supply chain system is:

$$\Pi_{S}^{C*}=\frac{1}{4}\left(\frac{4m_{0}{}^{2}}{m_{1}}-2c_{1}\alpha +\frac{\alpha^{2}}{\beta }+c_{1}{}^{2}\beta +4\mu m_{0}+m_{1}\mu^{2}\right).$$

**Proposition** **2.** *Under a centralized decision-making scenario, if the unit production cost of the new product, c_1_, increases, then the sale price of the product p will increase; if the unit production cost of the remanufactured product, c_2_ increases, and the direct recycling price of the waste product will decrease (Please see Appendix C for proof)*.

Proposition 2 shows that, in a centralized decision-making scenario, the manufacturing cost of the product affects the sale price of the product and its recycling price. As seen from this proposition, enterprises should make further use of the overall supply chain advantage under a centralized decision-making strategy. This is because supply chain efficiency can be enhanced, and the unit production cost of new products is reduced when combining electronically-equipped production that is unique in industry with advanced technical equipment in production activities that are accompanied by product patent, logistics synergy, and information sharing, etc. When recycling electronic wastes, related recycling enterprises should also adopt advanced technology to effectively handle waste products and lower the unit product cost in remanufacturing, increase cost savings, and expand the recycling scale to generate greater economic and environmental benefits.

**Proposition** **3.** *Under centralized decision-making, the profit level of the supply chain system is higher than in decentralized decision-making; the respective sale price of the product is lower than in decentralized decision-making, and the direct recycling price of waste products and recycling quantity are higher than in decentralized decision-making (Please see Appendix D for proof)*.

As seen in Proposition 3, compared to decentralized decision-making, consumers can buy their preferred electronic products at a lower price in a centralized supply chain decision-making model. Consumers can also sell their waste products at a higher price under a centralized decision-making scenario, whereby they can obtain greater benefits, and the profit is higher in the supply chain system with a better performance. This is due to the elimination of internal competition in the CLSC under centralized decision-making since supply chain members voluntarily make decisions based on profit maximization of the overall system. In such a case, the supply chain operates more efficiently while achieving higher profits. On the contrary, supply chain members in a decentralized decision-making scenario compete with each other internally. According to Stackelberg’s game theory, each player makes decisions based on their own profit maximization, neglecting the benefits of the overall system, which results in profit loss to the system as a whole. Therefore, centralized decision-making is preferred in the supply chain, although it is not practical, and the profit level achieved under centralized decision-making is only the maximum possible value. To optimize each party’s profits in decentralized decision-making and achieve a profit level comparable to other scenarios, each player in the supply chain should voluntarily participate in its profit coordination to facilitate both systemic and individual profit optimization while generating greater social and environmental benefits.

## 4. Revenue-and-Expense Sharing Contract Coordination Mechanism Design

There are two main objectives in supply chain coordination: (1) profit maximization for the supply chain system so that the coordinated system can achieve a profit level that is the same or close to the level generated in centralized decision-making, and optimize profit maximization of the supply chain when adopting decentralized decision-making; (2) profit maximization for all enterprise nodes in the supply chain so that each coordinated party will achieve a profit level that is higher than before coordination, thereby achieving Pareto improvement in profits for all nodes in the supply chain. The revenue-and-expense sharing contract coordination mechanism design is described below.

The manufacturer sells products to the retailer at a wholesale price w and pays a transfer price $B_{R},B_{E}$, to the retailer and third-party online recycling platform, respectively, for recycling electronic waste products; the retailer sells products to the consumer at a direct sale price. The manufacturer and retailer share the sale profits and recycling costs at 1 − $\gamma_{1}$ and $\gamma_{1}$, respectively, whereas the manufacturer and the third-party online recycling platform share the recycling cost incurred by the latter when recycling products. In this context, the respective equations for the profit coefficients of the manufacturer, retailer, and third-party online recycling platform, and overall profit coefficient transferred to the supply chain, are as follows:

Coordinated decision-making model:

(3) $$\begin{array}{c} {\max\Pi }_{M}^{SC}=\left(1-\gamma_{1}\right)\left(p\left(\alpha -\beta p\right)-\left(1+\phi \right)A_{R}q_{R}\right)+\left(1+\gamma_{2}\right)\left(\left(1+\phi \right)A_{E}q_{E}\right)\\ +w\left(\alpha -\beta p\right)-B_{R}q_{R}-B_{E}q_{E}\\ \max\Pi_{R}^{SC}=\gamma_{1}\left(p\left(\alpha -\beta p\right)-\left(1+\phi \right)A_{R}q_{R}\right)+B_{R}q_{R}-w\left(\alpha -\beta p\right)\\ \max\Pi_{E}^{SC}=\left(-\gamma_{2}\right)\left(\left(1+\phi \right)A_{E}q_{E}\right)+B_{E}q_{R}\\ \Pi_{S}^{SC}=\Pi_{M}^{SC}+\Pi_{R}^{SC}+\Pi_{E}^{SC} \end{array}$$

The decision-making behavior of each player is solved using reverse regression.

We solve the first-order partial derivative of p and $A_{R}$ in $\Pi_{R}^{SC}$, solve the first-order partial derivative of $A_{E}$ in $\Pi_{E}^{SC}$, subject to $\frac{\partial \Pi_{R}^{SC}}{\partial p}=0,\frac{\partial \Pi_{R}^{SC}}{\partial A_{R}}=0,\frac{\partial \Pi_{E}^{SC}}{\partial A_{E}}=0$, and obtain $p,\;A_{E},\;A_{R}$. We substitute p, $A_{R}$, and $A_{E}$ into $\Pi_{M}^{SC}$, solve the first-order partial derivative of w, $B_{R},\;\mathrm{and}\;B_{E}$ in $\Pi_{M}^{SC}$, and $\frac{\partial \Pi_{M}^{SC}}{\partial B_{R}}=0,\frac{\partial \Pi_{M}^{SC}}{\partial B_{E}}=0,\frac{\partial \Pi_{M}^{SC}}{\partial w}=0,$ and obtain: $w^{SC*}=\frac{\alpha \left(1-\gamma_{1}\right)}{3\beta },\;B_{R}{}^{SC*}=\frac{m_{0}\left(m_{1}+m_{2}\right)\left(-1+\gamma_{1}\right)N}{M},\;B_{E}{}^{SC*}=\frac{2m_{0}\left(-1+\gamma_{1}\right)\gamma_{2}S}{M},\;p^{SC*}=\frac{\alpha -\frac{1}{3}\alpha \left(-1+\gamma_{1}\right)-\alpha \gamma_{1}}{2\beta \left(-1+\gamma_{1}\right)},\;A_{R}{}^{SC*}=-\frac{m_{0}\left(8m_{1}{}^{3}\left(-1+\gamma_{1}\right)\left(-1+\gamma_{2}\right)+T\right)}{M\left(1+\phi \right)},\;A_{E}{}^{SC*}=\frac{2m_{0}\left(-1+\gamma_{1}\right)U}{M\left(1+\phi \right)}$.

Thus, the optimal profit of each player is:

$$\begin{array}{c} \Pi_{M}^{SC*}=\frac{\left(1-\gamma_{1}\right)K}{3\beta M},\;\Pi_{E}^{SC*}=\frac{{\left(-1+\gamma_{1}\right)}^{2}{I\gamma }_{2}}{M^{2}},\;\Pi_{R}^{SC*}=\frac{\alpha^{2}\left(-1+\gamma_{1}\right)}{9\beta }+\frac{HV}{M^{2}}+\left(1-\gamma_{1}\right)(\frac{2\alpha^{2}}{9\beta }+\\ \frac{m_{0}{}^{2}\left(m_{1}{}^{2}+3m_{1}m_{2}+2m_{2}{}^{2}\right)GV}{M^{2}}). \end{array}$$

**Proposition** **4.** *When revenue-and-expense sharing contracts are coordinated, $p=-\frac{\alpha -\frac{1}{3}\alpha \left(-1+\gamma_{1}\right)-\alpha \gamma 1}{2\beta \left(-1+\gamma_{1}\right)}$ is the retailer’s optimal selling price, and $A_{R}=-\frac{m_{0}\left(8m_{1}{}^{3}\left(-1+\gamma_{1}\right)\left(-1+\gamma_{2}\right)+T\right)}{M}$ is the retailer’s optimal direct recycling payment strategy. $w=-\frac{\alpha \left(-1+\gamma_{1}\right)}{3\beta }$, $B_{R}=\frac{m_{0}\left(m_{1}+m_{2}\right)\left(-1+\gamma_{1}\right)N\left(1+\phi \right)}{M}$ and $B_{E}=\frac{2m_{0}\left(-1+\gamma_{1}\right)\gamma_{2}S\left(1+\phi \right)}{M}$ is the manufacturer’s optimal transfer payment paid to the retailer and third-party platform, respectively, and $A_{E}$ = $\frac{2m_{0}\left(-1+\gamma_{1}\right)U}{M}$ is the third-party platform’s optimal pricing strategy (Please see Appendix E for proof)*.

Proposition 4 shows that when revenue-and-expense sharing contracts are coordinated, the optimal pricing strategies of each party are different compared to them before coordination. The most significant change is that the direct payment pricing is now affected by the sharing contract coordination coefficient as compared to being affected by consumer bargaining power without coordination. In addition, the manufacturer’s optimal transfer price is now affected by both the contract coefficient and the consumer bargaining power coefficient. The reason for this new phenomenon is due to the presence of the revenue-and-expense sharing contract, where the manufacturer has given up some profits. Since the manufacturer has shared some of the recycling fee incurred by retailers and the third-party platform, the impact of the consumer bargaining power on them in direct recycling has vanished. This is reflected in the direct recycling price paid by the retailer and online recycling platform, which are no longer affected by the consumer bargaining power coefficient. Moreover, due to the presence of the contractual relationship, the retailer, the online platform, and the manufacturer have formed a unified system from the consumers’ perspective. Therefore, when the manufacturer makes pricing decisions, they will consider consumer bargaining behavior when dealing directly with consumers. The higher the consumer bargaining power, the higher the recycling transfer payment that the manufacturer sets. This conclusion is expressed by the transfer payment equations $B_{R}=\frac{m_{0}\left(m_{1}+m_{2}\right)\left(-1+\gamma_{1}\right)N\left(1+\phi \right)}{M}$ and $B_{E}=\frac{2m_{0}\left(-1+\gamma_{1}\right)\gamma_{2}S\left(1+\phi \right)}{M}$.

By examining Proposition 4, we understand that, although consumers have low bargaining power in reality because of the presence of the pricing mechanism and the coordination strategy in the supply chain operation, and to promote consumers’ enthusiasm in recycling, consumer bargaining power is indeed considered to a certain extent in recycling pricing. Consumers participate in waste product recycling only as individuals, who, if given too much bargaining power, will not be conducive to the operation of the overall supply chain. Consumers neither have access to relevant scientific measures that are adopted by recycling enterprises to objectively measure the actual useful value of products, nor do they sacrifice their profits for the overall operational efficiency of the system identified from the integration level of the supply chain. On the contrary, product evaluation conducted by professional recycling enterprises can guarantee a certain level of impartiality. The establishment of an evaluation system for recycling products is urgently needed for China’s current recycling format of WEEE. With the establishment of a unified electronic waste evaluation system, product values can be estimated more scientifically while a consumer’s economic psychology and bargaining power can be controlled using binding legal regulations to strengthen a consumer’s recycling and environmental awareness.

## 5. Numerical Analysis

Based on the calculated results of the models, the sensitivity of coefficients and variables are analyzed. Through a numerical case study analysis, the responses of each party’s profit to different consumer bargaining power coefficients under the same set of parameters are studied, and different bargaining power coefficients are simulated to study the respective impact on M, R, E, and the supply chain system.

This study uses Mathematica when analyzing related parameter sensitivity. Based on the aforementioned assumptions and practical situations, parameter values are assigned as follows:

$$\gamma_{1}=0.4,\;\gamma_{2}=0.6,\;m_{0}=1000,\;m_{1}=2,\;m_{2}=1,\;\alpha =5000,\;\beta =2,\;\phi =0.1,\;\mu =1500,\;c_{1}=2000.$$

### 5.1. Sensitivity Analysis of the Consumer Bargaining Power Coefficient

When the consumer bargaining power coefficient is ϕ∈[0,1], a curved relationship figure is simulated using the recycling price, sale price, and affected profit level for each enterprise node.

**Figure 2:**
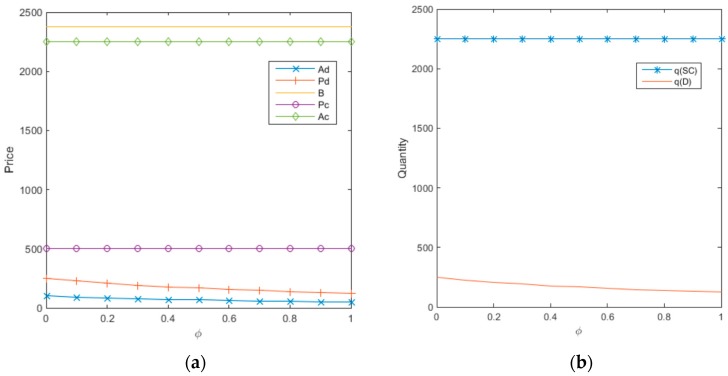
Sensitivity analysis of the consumer bargaining power coefficient to recycling price and quantity. (**a**) The recycling price; (**b**) The recycling quantity.

As shown in Figure 2a, under decentralized decision-making, consumers will attempt to exercise their bargaining power in recycling, hoping to raise the transaction price to a certain extent to achieve greater consumer welfare since the retailer and third-party online recycling platform have direct contact with consumers in electronic waste product recycling. However, the impact of the consumer bargaining power is very limited. In reality, consumers have very little bargaining power regardless of whether the direct recycling transaction is done online or off line, which is a consequence of an enterprise’s pursuit of profit maximization. Only when the transaction is transferred from online to offline will the consumer bargaining power have some impact, the extent to which is determined by the differences in estimating recycled product quality. Therefore, when the consumer unilaterally raises the price of recycled products, profits and efficiency in enterprises and the overall supply chain are lost, resulting in the downsizing of enterprises and a reduction in the final recycled product price, as shown in Figure 2 above.

In the sale, recycling, and remanufacturing of electronic products in a CLSC, manufacturers and retailers are the primary leaders. When there is no direct contact with consumers, the consumer’s initiative is lower. Therefore, the manufacturer’s decision regarding the final transfer payment is not affected by consumer bargaining power. When products are being sold, consumers do not have bargaining power as they do in recycling. The sale price of electronic products is usually determined by the retailer based on market supply and demand, and is not affected by consumer bargaining power.

The recycling quantity of waste products under revenue-and-expense sharing contract coordination is significantly higher than the recycling quantity under decentralized decision making, as shown in Figure 2b above. This is the external effect caused by lower internal competition when CLSC is coordinated. Due to the presence of contracts, supply chain members will voluntarily make their own decisions based on the new pricing strategy, making the operation of the supply chain more efficient with higher profits. Due to internal competition among supply chain members in decentralized decision-making, Stackelberg game theory holds true that each player in the supply chain will seek their own profit maximization in decision-making, neglecting the interest of the overall system, resulting in profit loss for the whole system. Each player in the supply chain should voluntarily participate in the profit coordination of the overall supply chain such that optimal profits will be achieved for both the overall supply chain and for each player, while creating greater social and environmental benefits. In reality, due to the individuality of consumers, even when the bargaining power of a particular consumer is strong, enterprises are still the market leaders. Therefore, unless consumers become a unified system, enterprises will only consider any room for bargaining in recycling payments, and consumer bargaining power behavior will not impact the industry size. This conclusion supports the phenomenon of low consumer bargaining power in real-life scenarios.

**Figure 3:**
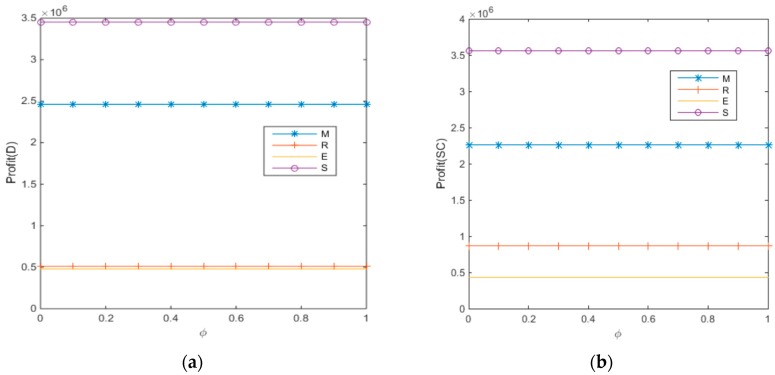
Impact of consumer bargaining power on each player’s profit. (**a**) The decentralized decision-making; (**b**) The centralized decision-making.

When consumer bargaining power is observed, revenue-and-expense sharing contracts are adopted to achieve the goal of profit coordination among enterprises in the supply chain to alleviate competition among internal members of the supply chain to reduce loss and optimize the profit of the overall supply chain, as illustrated by the profit increase in the overall supply chain in Figure 3a,b above. Sharing contracts among supply chain members strengthens the cooperation among all parties in the supply chain. To ensure profit optimization and strengthen cooperation among enterprises, the manufacturer, being the leader of the supply chain, will have to give up some profits. In this context, the bargaining power of consumers, who are paid for recycling electronic wastes in the supply chain as single individuals, is indeed quite insignificant in the unified enterprise system that plays the leader-role in the supply chain. This argument is supported by the changes in the trends of profit levels for each player and the supply chain illustrated in Figure 3a,b.

In reality, consumers do not have much bargaining power when recycling electronic waste, and we carried out a mathematical proof in this study to present a theoretical verification as follows. Since enterprises play a leader-role in the reverse logistics in a CLSC, a consumer’s direct recycling payment received from enterprises during recycling transactions is determined by the enterprises based on product quality, and as such, the consumers’ role is a “passive price recipient”. This explains why, in reality, consumers do not have much bargaining power in electronic waste recycling. The issue of consumer rights protection is becoming increasingly popular as we are living in an era of increased legal awareness. From the perspective of the overall supply chain, however, consumers should not possess too much bargaining power, otherwise the supply chain will malfunction or even stop functioning. As a rational economic agent, when a consumer receives a recycling payment, the price is a major factor, but not the only factor that affects consumers’ recycling behavior. Therefore, in order to promote consumers’ recycling behavior, we need to also adopt measures related to social responsibility, environmental awareness, and legality, in addition to solely relying on increasing consumer bargaining power.

### 5.2. Sensitivity Analysis of the Channel Competition Coefficient 

**Figure 4:**
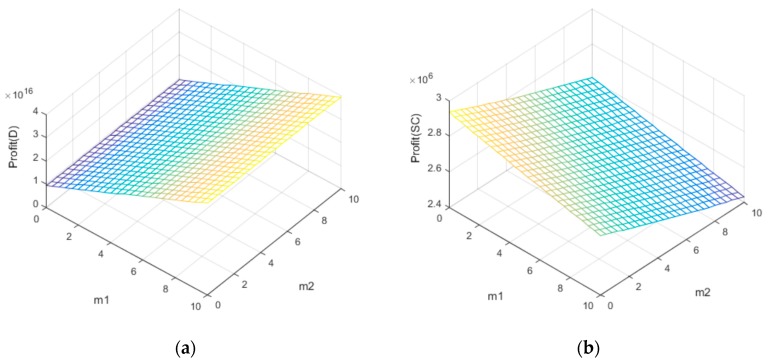
Sensitivity analysis of the channel competition coefficient. (**a**) The decentralized decision-making; (**b**) The centralized decision-making.

When analyzing the impact of the channel competition coefficient on the overall profit of the supply chain system, the values defined for all other parameters are kept the same as above, except for channel competition. As seen from Figure 4a,b, the optimal profits of the supply chain system before coordination are generated when approaching the interval endpoint. This indicates that when there is channel competition under a dual-channel mechanism, better recycling benefits can be achieved when there is only one channel leader, who is assisted by another channel in handling recycling. On the other hand, after supply chain contracts are coordinated, we can see intuitively from Figure 4b that the profit trends become gentler, displaying an upward trend as channel competition coefficients $m_{1}$ and $m_{2}$ increase. This is because the coordination mechanism has lowered the internal competition in the supply chain, increased coordination among all players, and indirectly reduced the competition intensity among different channels.

### 5.3. Sensitivity Analysis of the Revenue-and-Expense Sharing Contract Coefficient

**Figure 5:**
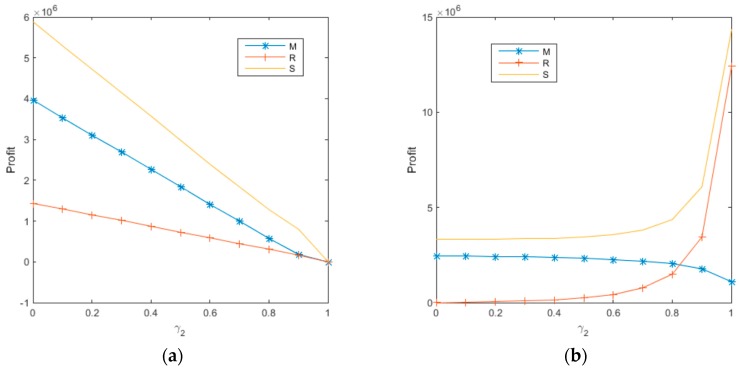
Sensitivity analysis of the contract coefficient. (**a**) The contract coefficient $\gamma_{1}$ γ−1; (**b**) The contract coefficient $\gamma_{2}$ γ−2.

Under revenue-and-expense sharing contract coordination in this study, the manufacturer and retailer share their profits from sales and recycling costs at $1-\gamma_{1}$ and $\gamma_{1}$, respectively, whereas the manufacturer and third-party online recycling platform share the recycling costs of waste and used products paid to consumers by the third-party platform at $1-\gamma_{2}$ and $\gamma_{2}$, respectively. The respective changes of the profit coefficient of the manufacturer, retailer, and third-party online recycling platform are shown in Figure 5a,b from the results of the sensitivity analysis on $\gamma_{1}$ and $\gamma_{2}$. An increase in $\gamma_{1}$ decreases the profits of the manufacturer and retailer, and when the sharing coefficient is equal to one, the profits of all parties are at a minimum. An increase in $\gamma_{2}$ enhances system profits and income of the third-party online recycling platform, leading to a decrease in the manufacturer’s income. This implies that, being the industry leader, the manufacturer will give up a certain degree of profits to motivate other members to improve the efficiency of the entire supply chain, but the coefficient of the revenue and expense sharing contract should be set within a reasonable range.

## 6. Conclusions

The primary research method adopted in this study is theoretical and includes a numerical case study. Following the orientation of the issue and combining it with practical scenarios, a dual-channel recycling pricing coordination mechanism was analyzed step-by-step and in detail, linking theory with practice. In terms of research content, this study is built on previous scholarly research and focused on consumers’ lower bargaining power in the existing recycling pricing coordination mechanism to propose that it has a positive impact on recycling pricing. Under such a condition, the study also presented the theoretical basis for the manufacturer in making price decisions and adopted a coordination mechanism in a CLSC. In terms of results, this study adopted the comparative statics analysis method. The research approach used the dual-channel assumptions to construct the model. Decision optimization was conducted for finding the best solution and the results were compared. Optimization and coordination theories such as game theory and operations research were the primary solutions and tools employed.

The innovation of this study lies in the introduction of consumers bargaining power into the waste and used product recycling model. Building on the logistics model of unifying the retailer and online recycling platform in recycling, the optimal pricing is generated under such a scenario that can motivate consumers to participate more actively in recycling waste products, adopting formal recycling behavior, and selecting proper channels. At the same time, the impact of the consumer bargaining power coefficient on electronic waste recycling is also incorporated into the dual-channel recycling model, extending the CLSC to include consumers, in addition to the major enterprise players at each node, which provides direction for subsequent research on consumer behavior in conjunction with the CLSC.

Due to assumptions that were made in this study, there are still areas in this study that need further investigation. First of all, the quality of the new and remanufactured products was assumed to be exactly the same in the present model, and the product demand coefficient relationship was expressed as linear. As related research advances, other conditions such as fuzzy demand, consumer product preference, or product heterogeneity may be considered when studying pricing mechanisms and coordination strategies related to consumer bargaining power. Second, this study did not consider either the quality differences in recycled products or the impact of the existing funding system and subsidy policies in the dual-channel CLSC model. As the recycling industry develops, government subsidies and differences in recycled product quality may be further studied.

## Figures and Tables

**Table 1:** Definition and description of symbols.

Symbol	Definition
*w*	Wholesale price of product
p	Retail price of product
$A_{i}$	Direct recycling price of waste paid by player *i* to i∈{R,E}
$B_{R}$	Transfer price received by retailer
$B_{E}$	Transfer price received by third-party online recycling platform
$\Pi_{j}^{i}$	Profits player *j* receives under dual-channel model, j∈{M,R,E}, i∈{D,C,SC}
$c_{1}$	Unit product cost using new materials
$c_{2}$	Unit product cost using materials disassembled from product recycling
$m_{0}$	Voluntary market recycling quantity when recycling price = 0
$m_{1}$	Channel competition coefficient
$m_{2}$	Price sensitivity coefficient
μ	Unit product cost savings from remanufacturing $\mu =c_{1}-c_{2}$
$q_{i}$	Player *i*’s market recycling quantity coefficient
A	Potential market size
β	Price impact factor of sale product
∅	Consumer bargaining power coefficient
$\gamma_{i}$	Coefficient of player *i*’s revenue-and-expense sharing contracts

## Appendix A

In order to simplify the expression of the optimal solution of the model, the symbols used in the paper are the following:

$$\begin{array}{c} M=m_{2}{}^{4}{\left(3-2\gamma_{1}\right)}^{2}+2m_{1}m_{2}{}^{3}\left(21+4\gamma_{1}{}^{2}+8\gamma_{1}\left(-3+\gamma_{2}\right)-8\gamma_{2}\right)+12m_{0}{}^{4}(-1\\ +\gamma_{1})\left(-1+\gamma_{2}\right)+48m_{1}{}^{3}m_{2}\left(-1+\gamma_{1}\right)\left(-1+\gamma_{2}\right)+m_{1}{}^{2}m_{0}{}^{2}(69\\ +4\gamma_{1}{}^{2}-56\gamma_{2}+8\gamma_{1}(-9+7\gamma_{2})) \end{array}$$

$$\begin{array}{c} N=4m_{1}{}^{2}\left(-1+\gamma_{1}\right)\left(-1+\gamma_{2}\right)+2m_{1}m_{2}\left(4-6\gamma_{2}+\gamma_{1}\left(-5+6\gamma_{2}\right)\right)\\ +m_{2}{}^{2}\left(3-8\gamma_{2}+\gamma_{1}\left(-6+8\gamma_{2}\right)\right) \end{array}$$

$$S=6m_{1}{}^{3}\gamma_{2}+m_{2}{}^{3}\left(3-6\gamma_{1}+4\gamma_{2}\right)+2m_{1}m_{2}{}^{2}\left(4-5\gamma_{1}+11\gamma_{2}\right)+2m_{1}{}^{2}m_{2}\left(2-2\gamma_{1}+11\gamma_{2}\right)$$

$$\begin{array}{c} T=m_{2}{}^{3}\left(9-8\gamma_{2}+2\gamma_{1}\left(-5+4\gamma_{2}\right)\right)+2m_{1}m_{2}{}^{2}\left(14-18\gamma_{2}+3\gamma_{1}\left(-5+6\gamma_{2}\right)\right)\\ +m_{1}{}^{2}m_{2}\left(27-32\gamma_{2}+4\gamma_{1}\left(-7+8\gamma_{2}\right)\right) \end{array}$$

$$U=m_{1}{}^{2}m_{2}\left(9+2\gamma_{1}-22\gamma_{2}\right)+m_{1}m_{2}{}^{2}\left(9+4\gamma_{1}-22\gamma_{2}\right)+m_{1}{}^{3}\left(3-6\gamma_{2}\right)+m_{2}{}^{3}\left(3+2\gamma_{1}-4\gamma_{2}\right)$$

$$V=m_{2}{}^{2}\left(3-2\gamma_{1}\right)+4m_{1}{}^{2}\left(-1+\gamma_{1}\right)\left(-1+\gamma_{2}\right)+m_{1}m_{2}\left(7-8\gamma 2+\gamma_{1}\left(-6+8\gamma_{2}\right)\right)$$

$$\begin{array}{c} G=8m_{1}{}^{3}\left(-1+\gamma_{1}\right)\left(-1+\gamma_{2}\right)+m_{2}{}^{3}\left(9-8\gamma_{2}+2\gamma_{1}\left(-5+4\gamma_{2}\right)\right)+2m_{1}m_{2}{}^{2}(14-\\ 18\gamma_{2}+3\gamma_{1}\left(-5+6\gamma_{2}\right))+m_{1}{}^{2}m_{2}\left(27-32\gamma_{2}+4\gamma_{1}\left(-7+8\gamma_{2}\right)\right) \end{array}$$

$$\begin{array}{c} H=m_{0}{}^{2}{\left(m_{1}+m_{2}\right)}^{2}\left(m_{1}+2m_{2}\right)\left(-1+\gamma_{1}\right)(4m_{1}{}^{2}\left(-1+\gamma_{1}\right)\left(-1+\gamma_{2}\right)+2m_{1}m_{2}(4\\ -6\gamma_{2}+\gamma_{1}\left(-5+6\gamma_{2}\right))+m_{2}{}^{2}\left(3-8\gamma_{2}+\gamma_{1}\left(-6+8\gamma_{2}\right)\right) \end{array}$$

$$\begin{array}{c} K=3m_{1}{}^{3}\left(m_{0}{}^{2}\beta \left(1+4\gamma_{1}\left(-1+\gamma_{2}\right)-4\gamma_{2}\right)+16m_{2}\alpha^{2}\left(-1+\gamma_{1}\right)\left(-1+\gamma_{2}\right)\right)+\\ 12m_{1}{}^{4}\alpha^{2}\left(-1+\gamma_{1}\right)\left(-1+\gamma_{2}\right)+m_{2}{}^{3}(m_{2}\alpha^{2}{\left(3-2\gamma_{1}\right)}^{2}+24m_{0}{}^{2}\beta (-2\gamma_{2}+\gamma_{1}(-1+\\ 2\gamma_{2})))+m_{1}{}^{2}m_{2}(3m_{0}{}^{2}\beta \left(3-20\gamma_{2}+4\gamma_{1}\left(-4+5\gamma_{2}\right)\right)+m_{2}\alpha^{2}(69+4\gamma_{1}{}^{2}-56\gamma_{2}+\\ 8\gamma_{1}\left(-9+7\gamma_{2}\right)))+2m_{1}m_{2}{}^{2}(m_{2}\alpha^{2}\left(21+4\gamma_{1}{}^{2}+8\gamma_{1}\left(-3+\gamma_{2}\right)-8\gamma_{2}\right)+3m_{0}{}^{2}\beta (1-\\ 16\gamma_{2}+2\gamma_{2}\left(-5+8\gamma_{2}\right))) \end{array}$$

## Appendix B. Proof of Proposition 1

Hessian matrix $H=\left(\begin{array}{c} \frac{\partial^{2}\Pi_{R}^{D}}{\partial p^{2}}\frac{\partial^{2}\Pi_{R}^{D}}{\partial p\partial A_{R}}\\ \frac{\partial^{2}\Pi_{R}^{D}}{\partial A_{R}\partial p}\frac{\partial^{2}\Pi_{R}^{D}}{\partial A_{R}{}^{2}} \end{array}\right)=\left(\begin{array}{cc} -2\beta & 0\\ 0 & -2\left(m_{1}+m_{2}\right){\left(1+\emptyset \right)}^{2} \end{array}\right)$, $\left|H_{1}\right|=-2\beta <0,\;\left|H\right|=4\beta \left(m_{1}+m_{2}\right){\left(1+\emptyset \right)}^{2}>0$, where the retailer receives the maximum profit when $p=\frac{\alpha +\frac{1}{2}\left(\alpha +c_{1}\beta \right)}{2\beta }$, $A_{R}=\frac{-m_{0}\left(3m_{1}+m_{2}\right)+m_{1}\left(m_{1}+m_{2}\right)\mu }{2m_{1}\left(2m_{1}+m_{2}\right)\left(1+\emptyset \right)}$. Similarly, the Internet Recovery Platform achieves the largest target profits when $\frac{\partial^{2}\Pi_{T}^{D}}{\partial A_{E}{}^{2}}<0$, $A_{E}=\frac{-m_{0}\left(3m_{1}+m_{2}\right)+m_{1}\left(m_{1}+m_{2}\right)\mu }{2m_{1}\left(2m_{1}+m_{2}\right)\left(1+\emptyset \right)}$.

Proposition 1 is demonstrated.

## Appendix C. Proof of Proposition 2

Partial derivation of $p^{C*}$ and $c_{1}$ is $\frac{\partial \;p^{C*}}{\partial c_{1}}=\frac{1}{2}>0$, where the sales price $p^{C*}$ is a monotonously increasing function for the production cost $c_{1}$. That shows that the product sales price $p^{C*}$ will rise when the unit cost of new products $c_{1}$ increases.

$A^{*}$ partial derivative of the remanufactured product unit saved cost μ is $\frac{\partial A^{*}}{\partial \mu }=\frac{1}{2\left(1+\emptyset \right)}>0$. The direct recovery of waste products $A^{*}$ is a monotonously increasing function of the unit μ. That shows that the direct recovery price of the waste product $A^{*}$ will increase when the unit production saved cost μ of the remanufactured product rises. From $\mu =c_{1}-c_{2}$, we can know that when $c_{1}$ is fixed and $c_{2}$ is rising, the cost of remanufacturing μ is decreased. Similarly, the direct recovery price of the waste product $A^{*}$ will decrease when the unit production cost $c_{2}$ of the remanufactured product rise. Proposition 2 is demonstrated.

## Appendix D. Proof of Proposition 3

Subtracting the optimal solution of the model from the centralized decision-making and the decentralized decision-making:

$$p^{C*}-p^{D*}=\frac{\alpha +c_{1}\beta }{2\beta }-\frac{3\alpha +2c_{1}\beta }{4\beta }=\frac{c_{1}\beta -\alpha }{4\beta }<0,$$

$$A^{C*}-A^{D*}=\frac{-2m_{0}+m_{1}\mu }{2m_{1}\left(1+\emptyset \right)}-\frac{-m_{0}\left(3m_{1}+m_{2}\right)+m_{1}\left(m_{1}+m_{2}\right)\mu }{2m_{1}\left(2m_{1}+m_{2}\right)\left(1+\emptyset \right)},\;=\frac{-m_{0}\left(m_{1}+m_{2}\right)+m_{1}{}^{2}\mu }{2m_{1}\left(2m1+m_{2}\right)\left(1+\emptyset \right)}>0.$$

Proposition 3 is demonstrated.

## Appendix E. Proof of Proposition 4

Hessian matrix: $H=\left(\begin{array}{c} \frac{\partial^{2}\Pi_{R}^{SC}}{\partial p^{2}}\frac{\partial^{2}\Pi_{R}^{SC}}{\partial p\partial A_{R}}\\ \frac{\partial^{2}\Pi_{R}^{SC}}{\partial A_{R}\partial p}\frac{\partial^{2}\Pi_{R}^{SC}}{\partial A_{R}{}^{2}} \end{array}\right)=\left(\begin{array}{cc} -2\beta \left(1-\gamma_{1}\right) & 0\\ 0 & -2\left(1-\gamma_{1}\right)\left(m_{1}+m_{2}\right){\left(1+\emptyset \right)}^{2} \end{array}\right)$, $\left|H_{1}\right|=-2\beta \left(1-\gamma_{1}\right)<0,\left|H\right|=4\beta \left(m_{1}+m_{2}\right){\left(1+\emptyset \right)}^{2}{\left(1-\gamma_{1}\right)}^{2}>0$, where the Hessian matrix is always negative. The retailer receives the maximum profit when $p=\frac{\alpha +\frac{1}{2}\left(\alpha +c_{1}\beta \right)}{2\beta }$, $A_{R}=\frac{-m_{0}\left(3m_{1}+m_{2}\right)+m_{1}\left(m_{1}+m_{2}\right)\mu }{2m_{1}\left(2m_{1}+m_{2}\right)\left(1+\emptyset \right)}$. Similarly, $\frac{\partial \Pi_{E}^{SC}}{\partial A_{E}}=0,\frac{\partial^{2}\Pi_{E}^{SC}}{\partial A_{E}{}^{2}}=-2\gamma_{2}\left(m_{1}+m_{2}\right){\left(1+\emptyset \right)}^{2}<0$, where the Internet Recovery Platform achieves the largest target profits when $A_{E}=\frac{-m_{0}\left(3m_{1}+m_{2}\right)+m_{1}\left(m_{1}+m_{2}\right)\mu }{2m_{1}\left(2m_{1}+m_{2}\right)\left(1+\emptyset \right)}$.
